# Consent, including advanced consent, of older adults to research in care homes: a qualitative study of stakeholders’ views in South Wales

**DOI:** 10.1186/1745-6215-14-247

**Published:** 2013-08-09

**Authors:** Fiona Wood, Hayley Prout, Antony Bayer, Donna Duncan, Jacqueline Nuttall, Kerenza Hood, Christopher C Butler

**Affiliations:** 1 Institute of Primary Care and Public Health, School of Medicine, Cardiff University, Neuadd Meirionydd, Heath Park, Cardiff CF14 4XNWales, UK; 2 South East Wales Trials Unit, School of Medicine, Cardiff University, Neuadd Meirionydd, Heath Park, Cardiff, CF14 4XNWales, UK

**Keywords:** Consent, Older adults, Qualitative research, Mental capacity, Ethics, Recruitment, Common infections

## Abstract

**Background:**

Care home residents, especially those lacking capacity to provide consent for themselves, are frequently excluded from research, thus limiting generalisability of study findings. We set out to explore stakeholders’ views about the ethical and practical challenges associated with recruiting care home residents into research studies.

**Methods:**

Qualitative individual interviews with care home residents (n = 14), their relatives (n = 14), and general practitioners (GPs) (n = 10), and focus groups (n = 2) with care home staff. Interviews focused on the issues of older adults consenting to research in care homes, including advanced consent, in general and through reference to a particular study on the use of probiotics to prevent Antibiotic Associated Diarrhoea. Data were analysed using a thematic approach incorporating themes that had been identified in advance, and themes derived from the data. Researchers discussed evidence for themes, and reached consensus on the final themes.

**Results:**

Respondents were generally accepting of low risk observational studies and slightly less accepting of low risk randomised trials of medicinal products. Although respondents identified some practical barriers to informed consent, consenting arrangements were considered workable. Residents and relatives varied in the amount of detail they wanted included in information sheets and consent discussions, but were generally satisfied that an advanced consent model was acceptable and appropriate. Opinions differed about what should happen should residents lose capacity during a research study.

**Conclusions:**

Research staff should be mindful of research guidance and ensure that they have obtained an appropriate level of informed consent without overwhelming the participant with unnecessary detail. For research involving medicinal products, research staff should also be more explicit when recruiting that consent is still valid should an older person lose capacity during a trial provided the individual did not previously state a wish to be withdrawn if they lose capacity, and provided they do not indicate objection or resistance after loss of capacity.

## Background

Older people are often excluded from research studies on the basis that their inclusion would present the research team with ethical or practical difficulties [1]. Such exclusion has been described as unjustifiable and scientifically flawed because it is inappropriate, and possibly dangerous, to apply the results of research to the management of older adults in general when the research excluded an important sub-group of older adults [2,3].

There are many ethical and practical challenges involved in conducting research with older people, which will have contributed to their under-representation in research studies. These challenges include difficulty in recruiting older people due to their physical and cognitive impairments, [4-7] obtaining consent, [7-12] responding to concerns from family members, [7,13,14] and higher rates of attrition from the studies [7,9,11,15]. On the other hand, fewer family and employment commitments of older people may facilitate their participation [2]. Additional challenges in conducting research within care homes include collecting data during the care homes’ busy schedule, along with issues of privacy (for example staff entering a resident’s room during an research interview, [12]) or poor staff compliance with the intervention and data protection protocols [7].

People in care homes (4% of those aged 75–84, and 17% of those aged over 85 in the UK), [16] most of whom have multiple physical and mental health and social care needs, have been particularly prejudiced in terms of opportunities to participate in research, and there have been recommendations for more studies to be conducted within care home settings [17]. Researchers have been given guidance on how to achieve this. For example, a toolkit developed by the National Institute of Health Research has been developed to assist researchers with best practice on preparing and conducting research in care homes [18]. Gaining informed consent can be a major challenge due to the high prevalence of dementia and consequent lack of mental capacity of many residents. The legal framework governing how consent is obtained also differs for different types of research study. Observational studies in the UK are governed by the Mental Capacity Act (2005), (section 30–34), which states that for those people who are deemed not to have capacity, a ‘personal consultee’ (somebody close to the person, but not acting in a paid capacity) is asked to give their *advice* on whether they believe the person would want to be involved [19]. Typically, the personal consultee is a relative or friend. Investigations of medicinal products are governed by the Medicines for Human Use (Clinical Trials) Regulations (2004), which state that *legal consent* to participate in the trial for those who lack capacity must be given by a Personal Legal Representative (family member, close friend) and, if such a person cannot be found, a Professional Legal Representative (doctor, solicitor, professional advocate), but not anyone involved in the study [20].

An advanced consent model has been advocated for patients who may not be able to give their consent to participation in a clinical trial at the time of randomisation [21]. Advanced consent may be particularly useful if the study intervention is administered in an emergency setting [22]. Other researchers have embedded qualitative sub-studies within trials to investigate participants’ perspectives of the trial recruitment processes [23-25]. To our knowledge there has been no previous research that has explored the views of residents, their relatives, or care home staff, about the potential, and problems, of conducting research within care homes, including issues of advanced consent and loss of capacity. Therefore, while conducting an observational study to assess prevalence of antibiotic prescribing and antibiotic associated diarrhoea in care homes (the Probiotics for Antibiotic Associated Diarrhoea (PAAD) study stage 1), we also conducted an embedded qualitative sub-study to investigate the views and experiences of residents, relatives, care home staff, and GPs about the practical and ethical challenges of conducting research (including trials of medicinal products) in care homes. More specifically, we were interested in the views of these stakeholders about advanced models of consent and what should happen if a study participant loses capacity during a trial of a medicinal product. Our aim was to generate ‘bottom up’ data from the perspectives of key people that would be involved in study implementation, to improve the setup and conduct of a subsequent low-risk randomised controlled trial (ISRCTN 79,548,440) of a probiotic versus a placebo given alongside antibiotics (PAAD stage 2) and to share such guidance with others conducting research in care homes. By ‘low-risk’ we refer to the core-set of risks inherent in the trial protocol which impact on the participants’ safety and rights. The risks associated with the IMP (Investigational Medicinal Product) (in our case a probiotic) should determine the trial procedures for monitoring the safety of patients [26]. In respect to the PAAD stage 2 study, by ‘advanced consent’ we refer to a situation where residents are recruited for a 12 month period, but only randomised to receiving a probiotic or placebo at the next point at which they are prescribed an antibiotic (which may be anything from the next day to 365 days after giving consent.

## Method

We used qualitative research methods because this would allow us to explore in-depth respondents’ views and experiences, including topics that we were unable to predict in advance. We thought it important to understand the views of a range of stakeholders and so our respondents included residents, relatives, care home staff, and GPs who have a responsibility for the general medical care of residents and who may be asked to assess eligibility for research studies. We recruited through 11 care homes in South Wales, which were participating in the PAAD Stage 1 study. Data collection was undertaken through a combination of face-to-face interviews with residents, relatives and GPs to facilitate in-depth reflection of respondents’ own involvement in research studies, and focus groups amongst care home staff to facilitate discussion about their collective involvement in research.

### Residents

Care home staff approached residents whom they felt had the mental capacity to consent to the qualitative sub-study. Residents were considered eligible to participate in the qualitative sub-study even if they had not given consent to participate in the PAAD stage 1 study. 14 residents consented to the study, all of whom had consented to take part in PAAD stage 1. Interviews were conducted in a private room within the care home (usually the resident’s bedroom) by a research nurse (HP). Interviews lasted between 9 and 54 minutes with an average of 23 minutes. Each resident was offered £25 in recognition of their time.

### Relatives

14 relatives of residents (4 partners and 10 sons or daughters) were also invited by the care home staff to be interviewed (all of whom had given advice as a personal consultee that their relative should be part of PAAD stage 1) and all consented. Interviews were conducted in a private room within the care home by a research nurse (HP). Interviews lasted between 12 and 31 minutes with an average of 19 minutes. Each relative was offered £25 in recognition of their time.

### Care home staff

Each care home was asked to nominate 3 members of staff who were most closely involved with the PAAD study. This staffs were invited to participate in a focus group that took place at a city centre hotel. 19 staff participated in the focus groups from 10 care homes. Two focus groups were held, one with senior staff (10 participants) and the other with junior staff (9 participants), and each group discussion lasted approximately 90 minutes. The focus groups were facilitated by a research nurse (HP) and a qualitative researcher (FW), both of whom were experienced in running focus groups but neither of whom had had prior contact with the care home staff regarding the PAAD study. Since care home staff were participating ‘off duty’, £25 was offered to each participant. Lunch was provided, and travel expenses were reimbursed.

### General Practitioners (GPs)

Senior care home staff were asked to name the main GP who attended patients within the care home. Letters of invitation were sent to 11 GPs and two GPs responded. In view of this, the researchers directly contacted sixty-nine other GPs in the Health Board area to request an interview, which resulted in 10 GPs agreeing to take part in face-to-face interviews. (Three of these GPs were aligned to a care home participating in PAAD Stage 1, while seven were not, although nevertheless attended people in care homes). GP interviews lasted between 20 minutes and 35 minutes with an average of 27 minutes. GPs were offered £50 for their time.

### Data collection

An interview topic guide defined the main topics whilst allowing flexibility to pursue issues in more depth as they emerged from the interviews and focus groups (see Additional files 1,2,3 and 4). Broad subject areas included their views of the consent processes that had been used for PAAD stage one and their experiences of participating in PAAD stage one, where appropriate. We collected data on the various merits and problems associated with a number of models of consent that could be used for a trial that lasts a reasonably long period such as 12 months (see Additional file 5). Discussion also covered how consent discussions should take place (for example, with a witness, over the phone, in person, by post). Respondents were asked what time frame they felt advanced consent should cover. In addition, the researcher presented the respondent with a range of hypothetical scenarios about taking advanced consent and asked the respondents to reflect on the potential advantages and disadvantages. They were also asked for their opinion on what should happen should the resident lose (and potentially regain) capacity during a research trial. Data from the focus groups and interviews were audio-recorded and transcribed verbatim.

### Data analysis

Data were analysed using thematic analysis with an abductive approach (incorporating themes that had been identified in advance and themes that were derived from the data) [27]. This approach involves systematically coding data according to a thematic framework, which is developed iteratively. Researchers met regularly to compare coding, discussing evidence for themes, and came to a consensus on the final framework. The thematic framework was applied to the data using the coding software package NVivo 8 [28]. Interpretations were discussed between all authors.

### Ethical approval

South East Wales Research Ethics Committee approved the study (10/WSE03/31). All potential respondents were provided with an information sheet and all respondents gave written informed consent prior to data collection.

## Results

Four main ethical topics emerged from the data. These were: 1) respondents’ assessments of harms and benefits of participating in the observational study and the proposed trial; 2) the process of gaining informed consent; 3) views about models of advanced consent; and 4) what should happen when residents lose capacity during a trial. Data extracts, including the respondent identification number, are used to illustrate the themes.

### Respondents’ assessments of the harms and benefits of participation in research studies

Respondents generally had few concerns about low-risk observational studies. Residents reported that they wanted to participate in the PAAD study as it was an interesting topic, and they wanted to participate altruistically on the basis of helping science and reducing diarrhoea in their care home. A few residents who had consented to the PAAD stage 1 study subsequently withdrew, and care home staff suggested that these residents might not have fully understood the study when consent was given. “*I think that’s why they’ve withdrawn, because they didn’t fully understand at the beginning, or, they may have forgotten, just because of, you know, slight memory problems”*. (Focus Group, senior staff).

Similarly relatives felt that the PAAD study was “*relatively harmless*” and could see the potential benefits to medicine and society *“well, any advance in science is worthwhile”* (Interview, Relative 2), as well as the potential benefits to their family member in the care home “*it’s for her benefit at the end, the long term isn’t it?”* (Interview, Relative 8). However, a few relatives did have some general concerns, for example whether the proposed intervention had been tested on healthy populations. While most relatives expressed full support for research conducted in care homes, a few stated they had concerns that residents were too elderly or frail and their participation in research was inappropriate (despite the residents sometimes themselves being mentally capable and having consented to take part).

Care home staff reported that one of their main reasons for participating in research generally was that older people, and care homes settings more specifically, had traditionally been neglected in medical research “*because there isn’t a lot of research in elderly care*, *we just felt that it*’*d be nice to do something that would be research based*” (Focus Group, junior staff). They also felt that there were professional benefits of being involved in university based research, supported by training, that would motivate the staff “*you need something to bring you up*, *bring you out*, *make sure that you*’*re still doing something with your career*, *if you like*, *and we did*, *we sat down at one of our meetings*, *for the qualified and we talked about it*, *and felt it would be good for us*, *stimulate us*.” (Focus Group, senior staff). Care home staff also felt that the PAAD study topic (antibiotics and diarrhoea) was an important issue for them. “*I think from our point of view*, *we see lots of antibiotics prescribed regularly*, *and all too often*, *not perhaps the correct antibiotic being prescribed*, *which does have the effect of diarrhoea*” (Focus group, senior staff).

Some GPs were concerned about how they, or their GP colleagues, would be financially reimbursed for their role in the PAAD Stage 2 study, but others reflected that the PAAD study would be clinically useful and would benefit GPs educationally, perhaps through increased awareness of antibiotic associated diarrhoea within care homes. “*Rates of C*.*diff or harm that comes from it* […]*I think you have to see it as something clinically useful*, *as something that is going to make a clinical difference in the end*’ (Interview, GP 8).

### The process of obtaining consent

Residents were generally supportive that relatives should be asked to act in the capacity of a personal consultee or a personal legal representative. Most felt that the relative would have a good idea of their wishes and that it would be better to ask a relative than a professional legal representative as the former would know the resident well. “*Oh yes*, *a relative*, *yes*, *because I think they know you better*, *and you understand them better*” (Interview, Resident 10). However, one resident doubted whether all relatives could be trusted to act in the best interests of residents “*you don*’*t know see*, *whether they*’*re* [*the family*] *all that trustworthy*” (Interview, Resident 9). Other residents felt that some relatives may not want to be bothered and may be too busy to help. Other potential problems included difficulties with relatives who live a long way from the care home, and some relatives themselves being mentally incapable.

Many relatives reported that they had wanted more details about the PAAD study in the information sheet (4 pages long), while others reported that they were overwhelmed with the paperwork, and wanted the information in a simple and short format. The relatives included in the sample for the qualitative sub-study were typically regular visitors at the homes. Many reported that they found their close relationship with the resident and with care home helpful when being asked to be a personal consultee. They felt they knew the residents’ wishes well: “*I think they* [*residents*] *trust their relative to act on their behalf*. *I think a stranger coming in and trying to talk them into doing something*, *they would be on their guard*” (Interview, Relative 12). It was felt that relatives who visit irregularly, or who have little contact with the home, would feel more anxious about being asked to take on the role of personal consultee or personal legal representative, partly because they would not be able to personally monitor the resident’s condition and partly because trust would be less well established between care home staff and relatives “*I would probably say no* [*to acting as a personal legal representative*] *if I was here only once a month to be honest with you*” (Interview, Relative 8).

Although the care home staff were motivated to participate in PAAD, most staff were surprised at the scale and intensity of the workload involved with implementing research and with the time required for consenting patients “*it*’*s also finding the time to do the consenting*, *and spend that extra bit of time to talk through*, ‘*cos we*’*re getting them interested in it*, *you know*, *um*, *well we did find that a bit hard*, *just getting the time and really sit down*, ‘*cos with 54 residents*, *and getting the relatives in*, *a lot of them would come in after tea*-*time*, *which is a really busy time*, *you know around tea*-*time*, *after tea*-*time*, *a really busy time*, *only two qualified on*, *quite hard then*” (Focus group, senior staff). Focus group participants reported that senior staff were better able to seek consent from relatives than junior staff. Specific difficulties included: i) communicating with relatives who visited infrequently, ii) locating a nominated consultee when no relative could be found, and iii) explaining the study to residents considered to have capacity, only for the resident to forget the purpose of the study afterwards. Some staff reported that relatives wanted simple explanations and were slightly put off by the length of the information sheet: “*I think the way it will be written down will make a lot of difference as to whether people consent or not because*, *you know*, *a lot of words can sometimes put people off*, *if it*’*s a lot of terminology*” (Focus group, junior staff). Simplified information sheets, which had been designed for residents, were consequently sometimes used with relatives.

Some GPs reflected on the difficulties in obtaining consent from older people with cognitive impairments. They had concerns that residents would forget important details about the study, even during the time they were given to reflect on their participation. In view of this, some queried whether truly informed consent could ever be obtained. The majority of GPs felt that most relatives would be happy to be a personal consultee or a personal legal representative and relatives would consent to the PAAD study since it was a “*benign study*” (Interview, GP 08). However one GP felt uncomfortable including patients without capacity in a trial on the basis of another’s advice “*I don*’*t feel comfortable about it at all because it*, *partly because I*, *I do feel that the patient should be able to give informed consent if they are receiving something that is out of the ordinary from their usual treatment*” (Interview, GP 5). Another GP stated that relatives may not always act in the resident’s best interests, “*I*’*m not always convinced that relatives act in the patient*’*s best interests*” (Interview, GP 4). Moreover, a few GPs felt that some relatives would not want to take on this responsibility and some may also be unable to retain or understand the information, “*it*’*s possible that the relative might be very elderly as well*, *and maybe losing their memory too*” (Interview, GP 2).

### Views about models of advanced consent

All but two of the residents stated that they would be happy to be consented just once to a 12 month study and it was not necessary to keep checking throughout the year to see if they still wanted to be involved “*once in the beginning*, *I think that would be sufficient*” (Interview, Resident 9). However, residents wanted to ensure that they could withdraw during the study “*I think um*, *there should be um*, *an escape route if you like*” (Interview, Resident 12). Other respondents suggested that their doctor should check every few months to ensure that they were well enough to participate in the study. In this respect, they wanted to be checked for eligibility rather than for consent.

None of the 14 relatives we interviewed expressed any major concerns about obtaining advanced consent for a study lasting 12 months. Generally they understood why obtaining advanced consent was required, although two relatives commented that 12 months can be an important time span, with residents’ health deteriorating considerably in this time. Relatives’ opinions on whether the research team needed to re-consent during the 12 months were divided. Three relatives felt that consent needed to be checked at regular intervals (typically every 3–6 months), although none of these relatives felt that this re-consent needed to be established in writing. Four relatives felt that it was not necessary to re-consent but that the care home staff should continue to remind relatives informally that the resident was still enrolled in the study “*well*, *if you*’*ve given consent*, *it might be nice to be reminded of it*, *you know*” (Interview, Relative 13). Seven relatives stated that re-consenting during a 12-month study was not required, as long as it was made clear that residents and relatives had the right to withdraw from the study.

Care home staff did not see a problem with using a model of advanced consent as they regularly used this method to gain consent for activities such as taking photographs within the care home or taking residents on outings. Care home staff thought that residents, or their legal representatives, should be reminded of their participation at the point of randomisation.

The majority of GPs felt that re-consenting during the course of the year would be unnecessary paperwork. One GP also raised the issue of how this could be managed if trial participants were to lose capacity during the study period “*I suppose the issue would be for someone who went from having capacity to losing capacity*, *and if you*’*re going to do*, *re*-*consent*, *you*’*re going to have to re*-*consent everybody*, *because it ought to be consistent*.” (Interview, GP1) However, some GPs did comment that it would be appropriate, and a matter of courtesy, to verbally remind residents (or relatives in cases where residents do not have capacity) at 3–4 month intervals that they are still part of the study, and are free to withdraw at any time.

### What should happen if a resident loses mental capacity during the trial?

Mental capacity relates to the ability of a person to make a specific decision at a particular time. For the PAAD study, senior care home staff were tasked with assessing whether residents had capacity to give consent for themselves or whether a personal consultee would need to be approached for their advice. PAAD stage 2 is planned as a 12 month study. Consequently, residents may consent in month 1, but could be randomised to either probiotic or placebo at any time between month 1 and month 12 depending on when they are next prescribed an antibiotic. It is possible that, if they were assessed for mental capacity at the point of randomisation, they would no longer be deemed as having mental capacity for the decision of whether or not to participate in the PAAD study. Residents were divided on whether they should continue to participate in the study without further consent from a relative if they lost mental capacity during the study. Some felt that if they lost mental capacity during that time they would want to continue regardless, “*I would say exceptionally*, *I would want to proceed with the survey* [*research study*], *because it*’*s so important*” (Interview, Resident 12). However, some felt that the resident would need reassessment by a health professional, “*I*’*d want you to check with my doctor to make certain it wasn*’*t doing any harm*, *because if my mental facility was going*, *I might be*, *it might be caused through that* [*the study drug*]” (Interview, Resident 3). Opinions were divided on whether to give this decision to the relative, “*the people doing the*, *er*, *research*, *I think they should really ask whoever is acting on their behalf*” (Interview, Resident 13), or the GP “*I think it must depend on the doctor*, *the GP who is in touch with the resident*” (Interview, Resident 12).

Relatives also differed in their opinions of this issue. Five relatives felt that a relative should be consulted about the resident’s continued participation “*you*’*re going to want somebody to act on his behalf*” (Interview, Relative 1), while four others felt that a relative should just be informed, as a matter of courtesy, that the resident had given their consent to participate in the study “*you know maybe it would just be like an act of common courtesy*” (Interview, Relative 14). However, five relatives felt that the resident should continue in the study regardless of whether a relative is informed or has given their consent. Many of the relatives acknowledged that this was a difficult issue. Some argued that relatives might have very strong views about this, but none speculated what might happen should a relative disagree with a resident’s continued participation.

With the exception of only a couple of individuals, all members of staff felt that if a resident loses capacity during a trial, then a legal representative would need to be approached and consent taken. When it was explained that, legally, this was not the case, some care home staff still insisted that they would still want to do this to “*cover themselves*”. At an absolute minimum, they felt that they should inform the relatives. “*Well*, *I think you*’*d have to re*-*consent with somebody that could give*, *well I feel I*’*d have to ask the next of kin*, *if we could carry on*, *I really do feel I*’*d have to do that*.” (Focus Group, senior staff).

With the exception of one GP, who said that he felt relatives should be approached for consent should a resident lose capacity, all felt that a resident should be allowed to continue in the trial. Most GPs said that it would be appropriate to inform the next of kin that the resident had consented in the study, but (with the exception of the one GP previously mentioned) no GP suggested that the relative could over-ride the resident’s wishes. The fact that residents could lose (and regain) capacity during the study supported their arguments that residents did not need to be re-consented during a trial lasting 12 months, as it would be impossible to re-consent those who had originally given consent for themselves and then lost capacity. They argued that if residents were to re-consent, we would need to do this consistently across the study population regardless of whether or not they had lost (or gained) capacity.

## Discussion

This qualitative sub-study about consent and recruitment of care home residents to research found that residents, relatives, care home staff and GPs are generally supportive of older adults in care homes participating in research studies. However, respondents were concerned about the best way of facilitating this, and some subtle, but important differences of opinion in how older adults should be recruited into studies were apparent.

One important issue relates to the comprehension and retention of study information to achieve an optimal level of informed consent. Residents and relatives were generally accepting of studies which they believed to be low risk and answered important questions that were in the interests of care home residents and the care home. However, some residents and relatives struggled to make sense of the purpose of the research and what was being asked of them. Previous research has shown that an accurate and comprehensive Participant Information Sheet (PIS) is not enough to ensure comprehension of the main issues [29]. In our study this was evidenced by some residents withdrawing from the study after initial consent, and relatives struggling with long detailed information sheets which sometimes care home staff supplemented, or substituted, with the short, graphically illustrated, information sheet which had been designed for residents. The data from our qualitative sub-study therefore confirm the importance of researchers and ethics committees making every effort to improve the quality of consent discussions and consent documents, ensuring use of familiar words and ideas, short sentences and paragraphs and routinely computing the reading ease of information sheets [30].

The comprehension and retention of information by residents and their relatives becomes even more of a challenge in a study which lasts a reasonably long period of time (12 months in our case), and when some relatives, asked to act in the capacity of a personal consultee or a personal legal representative, are themselves frail and at risk of becoming cognitively impaired. However, these specific challenges, and challenges raised by other researchers, are not a reason to exclude older adults from research. While it is important not to underestimate the difficulties, recruitment strategies need to be developed in order to maximise the involvement of frail older people while at the same time protecting their right to decline participation. While PISs are highly standardised, those who recruit older adults to research studies will need training in methods of communication which promote collaborative decision making and enable them to convey complex principles such as equipoise and randomisation in appropriate language. Recruitment of older people, both with and without capacity, to research studies can be complex but the awareness and integrity of the researcher is fundamental to maintaining the principle of non-exploitation [5]. Particular attention should be given to assessing visual and auditory and mental status, compensating for communication and sensory deficits through the use of innovative consent procedures such as pictorial information sheets and interactive tools that help researchers to assess the potential participants’ understanding and retention of information relevant to the study [8]. The second main issue relates to the role of personal consultee, or personal legal representative, particularly when a research subject loses mental capacity during a research study or trial. Some relatives expressed the view that the person they were related to was too elderly or frail and their participation in research was inappropriate, despite the resident themselves having had capacity to decide that they wanted to take part. If these relatives were to act as a personal consultee or legal representative it is uncertain whether their advice or decision on inclusion would reliably reflect the resident’s wish rather than their own. This was indeed raised as a concern by one resident. It is noteworthy also that one of the 10 GPs interviewed did not agree with including adults without capacity into any trial of a medicinal product. It is perhaps worth exploring the extent of this attitude amongst a larger and broader sample of GPs, health professionals, and care home staff, as if this belief is widely held it is likely that it will be a barrier to improving recruitment of this under-represented group into research trials, despite their heavy use of a wide range of health care technologies.

In a study that lasts 12 months, it is likely that some care home residents who originally have capacity to give consent, may lose mental capacity. Furthermore it is not unusual in such an age group for capacity to be temporarily lost, for example due to delirium associated with a urinary tract or chest infection [31]. One important finding of our study was that many staff, relatives and residents felt that if a resident loses capacity during a clinical trial, then a legal representative must be consulted about their continued participation in the study. This is in contrast to the majority of GPs who believed that this would not need to be the case. The Mental Capacity Act makes provisions for a personal consultee to advise on the continued participation of the research subject in studies, [32] but this is in contrast to clinical trials of medicinal products, where consent from an adult to participate in the trial remains valid, even after loss of capacity, provided the trial is not significantly altered.

### Limitations of the study

While this qualitative sub-study incorporates views from a wide range of stakeholders, we acknowledge that our data may be biased. For example, we were only able to interview relatives who were regular visitors to the care homes, residents who had capacity, and care home staff who were interested in participating in research. Although residents and relatives who had declined their consent to participate in PAAD stage 1 were invited to participate in the sub-study, only those residents and relatives who had agreed to participate in the main PAAD stage 1 study agreed to participate in the sub-study. This could have resulted in a biased sample that included more people who were more accepting of research. We also do not know how many residents were approached by care home staff to participate and declined to be interviewed, which may be a source of potential bias.

Furthermore the GPs who agreed to be interviewed may have been more knowledgeable of research ethics. We also acknowledge that our respondents may not have been so supportive of research had the trial within which we embedded our research been perceived as more harmful. In our analysis we have tried to reflect general issues about the ethics and practical issues of conducting trials in care home settings, rather than focus on the specifics of the PAAD study. This qualitative sub-study was undertaken in care homes in South Wales, and further work could be undertaken to further explore whether these issues remain relevant in other settings and amongst other populations.

## Conclusion and recommendations

Although care home environments pose practical problems to researchers, our findings should inform other research teams that care home residents, relatives and staff may be generally very supportive of participating in research. Given that older people are the heaviest consumers of health and social care, challenging ageist practices and attitudes in research is important if they are to gain maximum benefit from advances in understanding and management. Ethics committees are also in a strong position to influence research practice and to reduce unethical discrimination on the grounds of age or social situation, by challenging researchers who continue to exclude these groups from research.

Research teams should consider using models of advanced consent for trials of interventions where there is a high likelihood that participants may not have capacity at the point of randomisation. We would also recommend that researchers who are conducting trials of medicinal products in care home settings ensure that care home staff and residents are made aware that residents who have consented themselves to a study have the legal right to remain in the trial if they lose capacity during the study period, provided the person has not indicated a different choice.

## Competing interest

The authors declare that they have no competing interests.

## Authors’ contributions

FW and HP collected and analysed the data and drafted the manuscript. JN, DD helped with recruitment and the co-ordination of the study. AB, DD, KH, CCB participated in the design of the study and helped to draft the manuscript. KH and CCB conceived of the study. All authors read and approved the final manuscript.

## Supplementary Material

Additional file 1 Interview Schedule – Residents. Click here for file

Additional file 2 Interview Schedule – Relatives. Click here for file

Additional file 3 Focus Group Schedule – Care Home Staff. Click here for file

Additional file 4 Interview Schedule – GPs. Click here for file

Additional file 5 Models of consent discussed during interviews and focus groups. Click here for file
