# Supplementary material for: Consent, including advanced consent, of older adults to research in care homes: a qualitative study of stakeholders’ views in South Wales
Source: Trials. 2013 Aug 9;14:247. doi: 10.1186/1745-6215-14-247 (PMC3750808; doi:10.1186/1745-6215-14-247)
Supplement: Additional file 1 — Interview Schedule – Residents. [file 1745-6215-14-247-S1.doc]

Figure 1. **Interview Schedule Residents**

Can you start by telling me why you were (not) interested in participating in the PAAD study (stage 1)?

Some other residents within the care home may not have the mental capacity to fully understand the study. In such cases we refer the decision on whether they should participate to their relative. What do you think may be some of the benefits or some of the problems with asking a relative about this?

The PAAD study (stage 2) lasts for 12 months. Do you have any thoughts about participating in a research study which will last 12 months?

Although somebody might decide to give consent to participate at the beginning of the study, do you think we should ask them again during the study to check that they are still happy to participate? If yes, how often? how should this be done (over the phone, in person?),

Although a resident may be well enough to give consent to participate at the beginning of a study, it is possible that during the study their health deteriorates and they become confused. If this happened to you, would you want to continue your participation in the study?

Do you have any other thoughts about how care home residents are recruited into research studies?

Do you have any further thoughts generally about the PAAD study? (prompt: recruitment, impact on the home / staff / residents, how results will be used etc.)
