# Supplementary material for: Consent, including advanced consent, of older adults to research in care homes: a qualitative study of stakeholders’ views in South Wales
Source: Trials. 2013 Aug 9;14:247. doi: 10.1186/1745-6215-14-247 (PMC3750808; doi:10.1186/1745-6215-14-247)
Supplement: Additional file 2 — Interview Schedule – Relatives. [file 1745-6215-14-247-S2.doc]

Figure 2. **Interview Schedule – Relatives**

Can you start by telling me why you gave your advice that you thought your relative (the resident) would want to participate in the PAAD study?

How did you feel taking on this role?

Do you think there could be any problems with relatives taking a role of ‘personal consultee’?

The PAAD study (stage 2) lasts for 12 months. Do you have any thoughts about your relative’s participation in a research study which will last 12 months?

Because stage 2 is a trial of a medical product (a probiotic) alongside an antibiotic, if residents do not have capacity to consent themselves, we are required to gain consent from a legal representative who may be a relative. Do you think there could be any problems with relatives taking on the role of legal representative?

Although the study lasts for 12 months, residents will only be randomised to a placebo or probiotic at the time of being prescribed an antibiotic - this could be anything from 1 week to 11 months after giving consent. Would you be happy about being asked to give your advice to consent for this kind of study? Would you be happy for your relative to participate in stage 2 of PAAD (randomized trial of medical product)?

Do you think we should check with residents and relatives during the 12 months whether they are happy with their continued participation? (prompt: if yes, how often? how should this consent be taken)

Imagine Mr Edwards is a resident in a care home and has been assessed as having capacity to consent himself for the PAAD study stage 2. However, six months later he loses capacity. There is still a likelihood that he will need antibiotics in the future. Would you have concerns that Mr Edwards should still be part of the study?

Do you routinely bring probiotics in for your relative to use? If yes, why?

Do you have any further thoughts generally about the PAAD study?
