# Supplementary material for: Consent, including advanced consent, of older adults to research in care homes: a qualitative study of stakeholders’ views in South Wales
Source: Trials. 2013 Aug 9;14:247. doi: 10.1186/1745-6215-14-247 (PMC3750808; doi:10.1186/1745-6215-14-247)
Supplement: Additional file 3 — Focus Group Schedule – Care Home Staff. [file 1745-6215-14-247-S3.doc]

Figure 3. **Focus Group Schedule – Care Home Staff**

Why you think your care home was interested in participating in the PAAD study?

What are your thoughts about the consenting process for PAAD stage 1? (prompts: assessing competence, requesting advice from relatives, residents’ and relatives’ views of the study, staff time and skills)

Could there any problems with relatives taking on the role of personal consultee for residents who do not have capacity?

Because stage 2 is a trial of a medical product (a probiotic) alongside an antibiotic, if residents do not have capacity to consent themselves, we are required to gain consent from their legal representative who may be their relative. Do you think there could be any problems with relatives taking on the role of legal representative?

PAAD stage 2 lasts for 12 months. Residents will be recruited at the start but only randomised to placebo or probiotic at the time of being prescribed an antibiotic - this could be anything from 1 week to 11 months after giving initial consent. Do you feel comfortable asking a resident or relative for this kind of advanced consent? Can you think of any other problems or benefits of this model of consent?

Do you think we should ask the resident or relative at regular intervals throughout the study to check that they are still happy to participate? (prompt: if yes, how often? how should this consent be taken eg verbally, over the phone, in writing?)

Imagine Mr Edwards is a resident in a care home and has been assessed as having capacity to consent himself for the PAAD study stage 2. However, six months later he loses capacity. There is still a likelihood that he will need antibiotics in the future. Would you have concerns that Mr Edwards should still be part of the study? What would your concerns be?

Do you think the research team have provided enough support to your care home whilst the PAAD study has been undertaken? (prompts, adequate training, timing, information provision, what could we have helped more with)

Do you think your home would want to participate in future research studies?

Do you have any further thoughts generally about the PAAD study? (impact on home / staff / residents, how results will be used).
