# Supplementary material for: Consent, including advanced consent, of older adults to research in care homes: a qualitative study of stakeholders’ views in South Wales
Source: Trials. 2013 Aug 9;14:247. doi: 10.1186/1745-6215-14-247 (PMC3750808; doi:10.1186/1745-6215-14-247)
Supplement: Additional file 4 — Interview Schedule – GPs. [file 1745-6215-14-247-S4.doc]

Figure 4. **Interview Schedule – GPs**

Can you start by telling me about any involvement you have had with the PAAD study (stage 1) so far?

Do you (would you) have any ethical or practical concerns about your patients’ participation in the PAAD study (stage 1)?

PAAD stage 2 lasts for 12 months. Residents will be recruited at the start but only randomised to placebo or probiotic at the time of being prescribed an antibiotic - this could be anything from 1 week to 11 months after giving initial consent. Do you think residents or their legal representatives, should give their consent just at the beginning of the study, or do you think the research team should check periodically that they still consent to participate. (prompt: if yes, why? how often? how should this consent be taken)

Can you imagine any circumstances in which you think relatives/advocates might have problems acting as a legal representative for a patient in relation to giving consent to a resident’s participation in a clinical trial?

Are you aware of any other practical or ethical challenges associated with recruiting care home residents who may or may not have capacity to consent into a study such as the PAAD study?
