# Supplementary material for: Consent, including advanced consent, of older adults to research in care homes: a qualitative study of stakeholders’ views in South Wales
Source: Trials. 2013 Aug 9;14:247. doi: 10.1186/1745-6215-14-247 (PMC3750808; doi:10.1186/1745-6215-14-247)
Supplement: Additional file 5 — Models of consent discussed during interviews and focus groups. [file 1745-6215-14-247-S5.doc]

Figure 5. **Models of consent discussed during interviews and focus groups**

| Advanced Consent: At the beginning of a study, taking written consent to be randomised at some point (in the event of being prescribed an antibiotic in this case) and the consent lasting throughout the trial regardless of potential loss of capacity during this time. |
| --- |
| Renegotiating advanced consent in writing at regular time periods, typically 3 months periods, during a trial. |
| Written consent at the start of a study and then verbal consent given by the resident or their legal representative (by telephone) every month/3 months thereafter. |
